# Prevalence, risk factors and quality of life impact of work-related musculoskeletal disorders among school teachers in Cairo, Egypt

**DOI:** 10.1186/s12889-022-14712-6

**Published:** 2022-12-03

**Authors:** Viviane Farid Fahmy, Mohamed Abdel Maguid Tolba Momen, Nayera Samy Mostafa, Mohamed Yehia Elawady

**Affiliations:** Department of Community, Environmental and Occupational Medicine, Faculty of Medicine, Ain Shams University, Abassia, Cairo, Egypt

**Keywords:** Egypt, Quality of life, Musculoskeletal diseases (MSDs), Prevalence, Risk factors,Teachers

## Abstract

**Background:**

School teachers constitute an occupational group which reported a high prevalence of work-related musculoskeletal disorders (WRMSDs). Different individual, occupational and psychosocial factors have been identified to influence the complex process of WRMSDs. WRMSDs represent an important and costly occupational health problem being responsible for a poor quality of life (QOL) of teachers**.** This study aimed to determine the prevalence, the risk factors, and the impact of WRMSDs on the QOL of teachers.

**Methods:**

310 full-time teachers from 15 public schools were surveyed using an interview questionnaire about their sociodemographic and occupational characteristics, the Nordic Musculoskeletal Questionnaire and the SF-36 Health Survey (SF-36).

**Results:**

Self-reported prevalence of WRMSDs at any body part over the past 12 months among teachers was 66.77%. Neck pain (56.1%) was the most prevalent WRMSD followed by shoulders (53.2%), low back (53.2%) and knees (50.6%) pain. Female gender, body mass index, the number of students per classroom, the number of classes per week, different adapted awkward postures and the lack of enough supervisor’s psychological support at work were among the risk factors positively associated with WRMSDs. WRMSDs had a negative impact on the physical and mental QOL of teachers with WRMSDs as reflected by their lower scores on all scales of the SF-36 compared to their counterparts without WRMSDs (*p* ˂ 0.05).

**Conclusion:**

WRMSDs were a highly prevalent problem among teachers in Cairo, Egypt and negatively influencing their physical and mental QOL. Different individual, occupational and psychosocial factors had been shown to be significant predictors for the occurrence of WRMSDs reflecting their complex nature and multifactorial etiology.

## Introduction

Work-related musculoskeletal disorders (WRMSDs) are now considered one of the most prevalent and costly occupational health problems in both developing and developed countries [1, 2]. They constitute the second highest occupational disease after the occupational mental diseases in many industrialized countries [3].

Musculoskeletal disorders (MSDs) are defined as inflammatory or degenerative conditions affecting the joints, muscles, bones, ligaments, tendons, peripheral nerves or supporting blood vessels [4]. They develop over time and manifest as musculoskeletal ache, pain or discomfort with consequent functional impairment [5]. Most WRMSDs are caused or aggravated either by specific work tasks or by the effects of the surrounding working environment [4].

The teaching profession is regarded as one of the high-risk occupations for developing WRMSDs and started to attract an increasing concern in the past few years after being long neglected [2, 6]. Recent epidemiological studies demonstrated that school teachers constituted an occupational group which reported a high prevalence of WRMSDs in the different body regions compared to other occupational populations [1, 2, 7–11]. The prevalence of self-reported WRMSDs among school teachers has been suggested to range between 39 and 95% [2].

The back, neck and upper limbs musculoskeletal problems were the most frequently reported WRMSDs by school teachers in many international studies. In China, the prevalence of neck and/or shoulder pain among school teachers was 49% while the prevalence of low back pain (LBP) was 46% [10]. In Turkey, the most prevalent musculoskeletal complaints among school teachers were LBP (74.9%), shoulder pain (55.9%) and neck pain (47.9%) [9]. In Botswana, the upper back, shoulder, and neck WRMSDs were reported at prevalence rates 52.6%, 52.5%, and 50.8% respectively [6]. In Egypt, the prevalence of WMSDs were 96% in the previous 12 months among preparatory school teachers. The neck and back (83.5%) were the most commonly affected body regions followed by the upper limb [12].

Many risk factors have been identified to influence the complex process of WRMSDs as sociodemographic, psychosocial and occupational characteristics of the teachers [11]. Sociodemographic variables like gender [1, 2, 6, 8–10, 12], age [8, 10, 11] and body mass index (BMI) [10–12] have been considered as associated factors with the development of WRMSDs among school teachers. Several studies reported that psychosocial factors like high work demands, low job control, perception of a high stress level at work, job dissatisfaction, monotonous work, lack of social support had a significant association with WRMSDs among school teachers [2, 13, 14].

The high prevalence of WRMSDs among school teachers can also be explained by their unique and wide variety of daily work tasks and duties performed repeatedly for prolonged hours without adequate rest intervals [7, 13]. The nature of the different teaching tasks involves a lot of uncomfortable physical activities such as the significant use of neck flexion position like during frequent reading, preparing lessons, marking assignments, grading examinations, working on computer and school administrative work, repetitive overhead writing on board with hands elevated above shoulder level, lifting heavy loads such as books, examination sheets and projectors, long hours standing up in an inappropriate way while teaching and supervising the students inside the classroom, sustained and improper sitting postures on tables for several hours, acquiring uncomfortable and awkward postures like bending or recurrent twisting as turning from the board to the classroom and back again resulting in constant stresses on their musculoskeletal system [1, 2, 5–11, 14, 15].

Presentation of WRMSDs ranges from just discomfort and mild pain up to a severe medical condition which requires medical help or absence and frequent sick leaves from work. Treatment and recovery of cases with chronic and persistent musculoskeletal pain (MSP) are usually unsatisfactory, functional impairment and permanent disability with subsequent decline in performance and reduced work productivity, loss of job or early ill health retirement and economic burden from considerable health care and compensations costs for the employers and the organizations may result. They are also responsible for a poorer and impaired quality of life (QOL) of school teachers [1, 2, 10, 14, 16].

There is limited available information on the prevalence and risk factors of WRMSDs among school teachers in Egypt and their impact on their QOL has not also been investigated sufficiently in the literature. Thus, a greater emphasis needs to be placed on raising the awareness of the relationship between WRMSDs and the teaching profession to alleviate their burden and their impact on teachers’ QOL and on the education system.

The current study aimed to measure the prevalence of WRMSDs among a sample of school teachers working in Cairo, Egypt, to identify the potential risk factors associated with WRMSDs among the study participants and to determine their impact on the QOL of the study participants. This knowledge will assist policy makers in developing and implementing cost effective preventive strategies targeting MSDs risk factors, to reduce these career-threatening injuries and to improve the musculoskeletal health and the overall QOL of school teachers especially in the developing countries.

## Subjects and Methods

### Study design, setting and duration

An analytical cross-sectional survey was carried out in public (both governmental and experimental) primary, preparatory and secondary schools in Cairo Governorate, the capital city of Egypt. The data collection was conducted over a period of six months between October 2019 and March 2020.

### Study population

Employed school teachers in public schools in Cairo constitute our study population. Teachers who agreed to participate in the study and met the following criteria: of both genders, being a full-time school teacher, had at least one year of teaching experience, worked only in public schools either governmental or experimental schools, taught any school level (kindergarten, primary, preparatory and secondary) and taught any scholastic subject were included in the study.

Teachers who were excluded from the study had one or more of the following criteria: above 50 years old to avoid the confounding effect of age-associated degenerative changes resulting from the physiological process of wear and tear of the osteoarticular system, the decline in muscle mass, the loss of connective tissue elasticity, the thinning of cartilage and the reduced ability of tissue healing in response to accumulated soft tissue damage and that all occur with aging on studying WRMSDs [1, 8, 10], school administration staff such as school directors and supervisors, had MSDs due to other causes not work-related such as congenital (scoliosis, kyphosis, lordosis), traumatic (fractures, accidents) or medical causes as autoimmune and inflammatory joint conditions (rheumatoid arthritis, systemic lupus erythematous, gout, ankylosing spondylitis), neurological deficits (nerve root pain, radicular pain, multiple sclerosis), endocrine diseases and osteoporosis, had undergone a previous musculoskeletal surgery, had a medical history of malignant tumors and pregnant female teachers.

### Sampling method

A multistage cluster sampling technique was used. First stage, Cairo Governorate is divided into four geographical sectors (North, East, Middle and South Cairo) by the ministry of Education. Three sectors (North, East and Middle Cairo) were randomly chosen by a simple random sample. Second stage, Cairo includes 32 educational administrations. Three administrations, namely El Zeitoun from North Cairo sector, El Nozha from East Cairo sector and El Waili from Middle Cairo sector, were selected by a simple random sampling technique from the list of educational administrations of each sector. Third stage, El Zeitoun includes 43 public schools, El Nozha includes 42 public schools and El Waili includes 44 public schools. Five public schools from each educational administration were randomly chosen resulting in a total of 15 schools, among which 8 were governmental and 7 were experimental. Finally, eligible school teachers working in the selected schools were invited to participate in the study.

### Sample size

The sample size was calculated using the following parameters: an estimated 12-months prevalence of WRMSDs among school teachers of 50% [17], a confidence level of 95% with an accepted range of variation between (43%—57%), a sample error of 5% and a design effect of 1.5. It was found that a sample size of 294 subjects would be adequate. The sample size was calculated using Epi Info 7 program.

A total of 365 school teachers were invited and only 310 of them agreed to participate in this survey resulting in a response rate of nearly 85%. The 310 teachers were recruited as follows: 120 from governmental schools and 190 from experimental schools, and 84 of them were from El Zeitoun, 125 from El Nozha and 101 from El Waili. The number of recruited teachers differs from one educational administration to another since schools have different number of employed teachers and the response rate also varies from one school to another.

### Study tools

A structured interview questionnaire was used. It consisted of four sections as follows:

- *Section I & II*

Section I surveyed the sociodemographic characteristics (age, gender, marital status) and the physical characteristics (self- reported height and weight) of the studied school teachers. Section II explored the occupational characteristics (years in teaching profession, number of students/class, number of classes/week, giving study groups after the end of the scholastic day, comfortableness of school furniture and different adapted awkward work-related postures) and the psychosocial (work overload, working under pressure of time, monotonous work, low job satisfaction, inadequate supervisor and colleagues’ psychological support) risk factors for the occurrence of WRMSDs among the studied school teachers.

The questions used to investigate the sociodemographic, occupational and psychosocial data were collected from the reviewed literature about the potential risk factors for WRMSDs and were also derived from the standardized Dutch Musculoskeletal Questionnaire (DMQ) [18].

- *Section III*

It evaluated the occurrence of WRMSDs among school teachers. An Arabic version of the Standardized Nordic musculoskeletal questionnaire (NMQ) was used [19]. The NMQ is a valid, reliable and sensitive screening tool commonly used in the epidemiological studies conducted in many countries to analyse the musculoskeletal symptoms regarding the prevalence in the different body parts in an occupational health context including the teaching profession [20–22]. NMQ is also commonly used since the physical examination is more costly, time consuming and difficult for large samples that’s why it is rarely used by the researchers [23]. In this study, the NMQ was translated to Arabic and back translated to English by two different nonmedical expert translators from Faculty of Al Alsun, Ain Shams university. Comparisons were made to ensure same phrasing and meaning of the questions by the researchers. The NMQ was also translated into Arabic and used in previous Saudi Arabian studies [11, 24, 25].

The NMQ consists of a diagram showing the posterior view of a human body divided into 9 clearly marked anatomical regions (neck, shoulders, upper back, lower back, elbows, wrists/hands, hips/thighs, knees, ankles/ feet), and a dichotomous (“yes” and “no”) question for each anatomical area. The question assessed the prevalence of self- perceived musculoskeletal symptoms by the respondents such as ache, pain or discomfort in the different body segments over the last 12-months. The prevalence rates were calculated based on the proportion of teachers who reported WRMSDs in any body part to the total number of the respondents.

The following operational definitions were adopted for the different outcomes:

- ***WRMSD:*** a musculoskeletal disorder was considered work-related if the teacher began to experience the musculoskeletal symptoms after being employed in the teaching profession and if those symptoms happened only during the working hours in relation to work tasks and not to any recent or past trauma, worsened at work and relieved during holidays.
- ***12-months WRMSD:*** was considered present if the respondent reported the occurrence of work-related musculoskeletal symptoms such as ache, pain, numbness, tingling or discomfort in any body region during the last 12 months.

The definitions for a self-reported WRMSD were adapted from different studies [9, 15, 17]. They were explained by the researcher to the teachers. Then, they were allowed to decide for themselves whether or not they suffer from a WRMSD based on the offered definitions and time frame. For further statistical analysis, teachers were classified into 2 groups based on their responses: group of “WRMSD cases” if the teacher reported a WRMSD in at least one body part during the past 12 months, and group of teachers “WRMSD free” if the teacher did not experience any WRMSD in the past 12 months.

- *Section IV*

It assessed the impact of WRMSDs on the health related QOL of the studied teachers by using the Arabic version of the RAND 36-item Short Form Health Survey (SF-36) questionnaire [26]. The SF-36 is a widely used instrument for measuring health related QOL perception. It consists of 36 Likert-type and binary (“yes” and “no”) questions grouped into eight health-related QOL scales: general health perception, physical functioning, role limitations due to physical problems, role limitations due to emotional problems, bodily pain, vitality, social functioning, and mental health. Further, it includes a question “health transition item” (HTI) to assess changes in a person’s health perception over time. All items were scored within a range of 0 (the worst condition) up 100 (the best condition) with higher scores indicating better QOL. The HTI is scored from 1 to 5, where low scores indicate improved health and high scores indicate reduced health.

### Statistical analysis

The collected data were revised for completeness and consistency, coded and entered into Microsoft Excel 2019. Then they were exported to and analysed by using the Statistical Package for Social Sciences (IBM SPSS Statistics, version 25) software for Windows. Data were presented and analysed according to the type of variables as follows:

- * Descriptive Statistics*

To summarize the data, mean and standard deviation were calculated for quantitative variables while frequencies and percentages were calculated for categorical variables. The BMI was calculated by dividing the weight (in kgs) by the square of the height (in meters). Measurements of weight and height were self-reported by the teachers. BMI was classified into four categories using WHO criteria as: underweight (BMI < 18.5 kg/m^2^), Normal/healthy weight (BMI = 18.5—24.9 kg/m^2^), overweight (BMI = 25—29.9 kg/m^2^) and obesity (BMI > 30 kg/m^2^) [27].

b. * Inferential Statistics*

Comparisons between the two groups of teachers who did and did not report WRMSDs over the last 12 months to detect the basic statistical associations between the independent and the dependent variables were initially evaluated using the independent sample t- test that assessed a statistically significant difference between two independent means of the two groups of teachers with and without WRMSDs, and the Pearson chi-square test that detected a statistically significant difference between categorical variables. The level of statistical significance adopted for this study was a two -sided *P*-value ≤ 0.05.

The prevalence ratios (PRs) as well as their corresponding upper and lower 95% confidence intervals (95% CI) were calculated using the robust Poisson regression model to determine the association of WRMSDs as a binary dependent variable with each of the sociodemographic, occupational and psychosocial potential risk factors for the development of WRMSDs as independent variables and to compare the magnitude of effects of various risk factors on occurrence of WRMSDs among school teachers.

## Results

A total of **310** school teachers participated in this cross-sectional study. They were recruited from 15 public schools as follows: 120 (38.7%) teachers from 8 governmental schools and 190 (61.3%) teachers from 7 experimental schools and belonging to 3 educational administrations in Cairo Governorate as follows: 84 (27.1%) teachers from Zeitoun (North Cairo), 125 (40.3%) from El Nozha (East Cairo), 101 (32.6%) from El Waili (Middle Cairo).

### Sociodemographic and occupational characteristics of school teachers

Overall, 205 (66.1%) female teachers and 105 (33.9%) male teachers participated in the study. Their mean age was 45.2 ± 5.1 years with age ranging from 27 to 50 years. The majority of the teachers were married (88.1%). As regards the self-reported physical characteristics of the study participants, the average height of the studied teachers was 166 ± 7.9 cm and ranging from 150 to 190 cm. Their average weight was 84 ± 17.1 kg and ranging from 45 to 217 kg. Their average BMI was 30.5 ± 6.1. Nearly one third of the teachers (33.9%) were overweight and half of them (48.7%) were obese (Table 1).

**Table 1:** Comparison between teachers with and without WRMSDs as regards their sociodemographic and self-reported physical characteristics

		**All Teachers** **(*N* = 310)**		**Without WRMSDs** **(*N* = 103)**		**With** **WRMSDs** **(*N* = 207)**		**Statistical** **Test**	***P*- value**	**PR** **(95%** **CI)**
		**No**	**%**	**No**	**%**	**No**	**%**			
**Age in years**	27 – 35	25	8.1	9	36	16	64	χ^2^ = 0.55	0.46	
	36 – 40	27	8.7	8	29.6	19	70.4			
	41 – 45	75	24.2	19	25.3	56	74.7			
	46 – 50	183	59	67	36.6	116	63.4			
			**Mean ± SD**					t-test = 0.37	0.71	
		45.2 ± 5.1		45.3 ± 5.4		45.1 ± 5				
**Gender**	Male	105	33.9	65	61.9	40	38.1	χ^2^ = 58.9	**˂0.001**	2.14 (1.67 – 2.75)
	Female	205	66.1	38	18.5	167	81.5			
**Marital Status**	Single	22	7.1	10	45.5	12	54.5	χ^2^ = 3.75	0.29	
	Married	273	88.1	88	32.2	185	67.8			
	Widow	7	2.3	1	14.3	6	85.7			
	Divorced	8	2.6	4	50	4	50			
**Body Mass Index**	Underweight	1	0.3	0	0	1	100	χ^2^ = 8.99	**0.003**	1.33 (1.04 – 1.7)
	Normal	53	17.1	23	43.4	30	56.6			
	Overweight	105	33.9	44	41.9	61	58.1			
	Obese	151	48.7	36	23.8	115	76.2			
		**Mean ± SD**						t-test = -2.61	**0.009**	
		30.5 ± 6.1		29.28 ± 5.2		31.17 ± 6.37				

^Boldface values indicate statistical significance (^^*P* < 0.05)^

^***χ***^^***2*** Chi square test, *PR* Prevalence ratio, *CI* Confidence interval, *SD* Standard Deviation^

The average working experience of the teachers in the teaching profession was 21.3 ± 5.9 years. Nearly two-thirds of the teachers (68.7%) had an average teaching experience of 20 to 30 years. About 79% of the teachers had over 35 students per class. The average number of students per classroom as reported by the teachers was 45.2 ± 10.7. Nearly half of the teachers (50.6%) had 20 or less classes in their weekly teaching schedule. The average number of classes taught by the teachers per week was 20.9 ± 6.5. Nearly one third of the teachers (32.3%) taught private study groups after the end of the scholastic day. For the opinion of the teachers as regards the school furniture, more than half of the teachers (57.1%) reported that it was uncomfortable (Table 2).

**Table 2:** Comparison between teachers with and without WRMSDs as regards their occupational characteristics and their adapted work- related postures

		**All Teachers** **(*N* = 310)**		**Without WRMSDs** **(*N* = 103)**		**With** **WRMSDs** **(*N* = 207)**		**Statistical** **Test**	***P*- value**	**PR** **(95%** **CI)**
		**No**	**%**	**No**	**%**	**No**	**%**			
**No of years in** **teaching profession**		**Mean ± SD**						t-test = -0.75	0.45	
		21.3 ± 5.9		20.97 ± 6.14		21.5 ± 5.75				
**No of students / classroom**	≤ 35 students / class	65	21	36	55.4	29	44.6	χ^2^ = 18.14	**˂0.001**	1.63 (1.23 – 2.16)
	> 35 students / class	245	79	67	27.3	178	72.7			
		**Mean ± SD**						t-test = -4.82	**˂0.001**	
		45.2 ± 10.7		41.21 ± 11.13		47.2 ± 10				
**No of classes** **per week**	≤ 20 classes / week	157	50.6	66	42	91	58	χ^2^ = 11.1	**0.001**	1.31 (1.11 – 1.54)
	> 20 classes / week	153	49.4	37	24.2	116	75.8			
		**Mean ± SD**						t-test = -2.18	**0.03**	
		20.9 ± 6.5		19.76 ± 6.01		21.46 ± 6.68				
**Giving study groups after** **the end of the** **scholastic day**	Yes	100	32.3	45	45	55	55	χ^2^ = 9.22	**0.002**	1.32 (1.08 – 1.6)
	No	210	67.7	58	27.6	152	72.4			
**The school furniture is comfortable**	Yes	133	42.9	46	34.6	87	65.4	χ^2^ = 0.19	0.66	
	No	177	57.1	57	32.2	120	67.8			
**Holding neck in a forward bent posture** **for long periods**	Yes	279	90	84	30.1	195	69.9	χ^2^ = 12.23	**˂0.001**	1.81 (1.15 – 2.83)
	No	31	10	19	61.3	12	38.7			
**Holding neck in a backward bent posture** **for long periods**	Yes	67	21.6	20	29.9	47	70.1	χ^2^ = 0.44	0.51	
	No	243	78.4	83	34.2	160	65.8			
**Working with hands above shoulder level** **for long periods**	Yes	186	60	51	27.4	135	72.6	χ^2^ = 7.07	**0.008**	1.25 (1.05 – 1.49)
	No	124	40	52	41.9	72	58.1			
**Holding wrist bent** **for long periods**	Yes	215	69.4	64	29.8	151	70.2	χ^2^ = 3.78	0.052	
	No	95	30.6	39	41.1	56	58.9			
**Standing** **for long periods**	Yes	208	67.1	60	28.8	148	71.2	χ^2^ = 5.47	**0.02**	1.23 (1.02 – 1.48)
	No	102	32.9	43	42.2	59	57.8			
**Sitting** **for long periods**	Yes	69	22.3	16	23.2	53	76.8	χ^2^ = 4.03	**0.04**	1.2 (1.02 – 1.41)
	No	241	77.7	87	36.1	154	63.9			
**Working in** **a bent posture** **for long periods**	Yes	218	70.3	61	28	157	72	χ^2^ = 9.11	**0.003**	1.33 (1.08 – 1.63)
	No	92	29.7	42	45.7	50	54.3			
**Working in a twisted posture for long periods**	Yes	106	34.2	28	26.4	78	73.6	χ^2^ = 3.37	0.07	
	No	204	65.8	75	36.8	129	63.2			

^Boldface values indicate statistical significance (^^*P* < 0.05)^

^***χ***^^***2*** Chi square test, *PR* Prevalence ratio, *CI* Confidence interval, *SD* Standard Deviation^

As regards the different occupational postures adapted by the studied teachers while performing their various teaching activities, almost all the teachers (90%) held their neck in a forward bent posture for long periods as during reading and marking and 21.6% of them held their neck in a backward bent posture for long periods as in board writing. 60% of the teachers worked with their hands raised above the shoulder level for prolonged periods as during writing on board. More than two thirds of the teachers (69.4%) held their wrist bent for long durations. Nearly two thirds of the teachers (69.4%) stood for long hours while performing their occupational activities. Nearly one quarter of the teachers (22.3%) reported that they sat for extended hours while working. 70.3% of the teachers worked in a bent posture for prolonged durations and 34.2% worked in a twisted posture for extended durations (Table 2).

### Prevalence of WRMSDs among school teachers

Regarding the prevalence of the self-reported WRMSDs by body regions among the studied teachers over the last 12-months, 207 teachers complained from WRMSDs at any body part over the past 12 months. Therefore, the overall self-reported prevalence of WRMSDs at any body part over the past 12 months was 66.77% among the studied teachers.

Neck pain was the most prevalent WRMSD in the last 12 months as it was reported by more than half of the teachers (56.1%). Shoulders, low back and knees pain constitute the second category of body regions with the highest reported prevalence rates after neck pain in the past 12 months. Shoulders and LBP were equally reported by more than half of the teachers (53.2%), and closely followed by knees pain (50.6%).

**Fig. 1:**
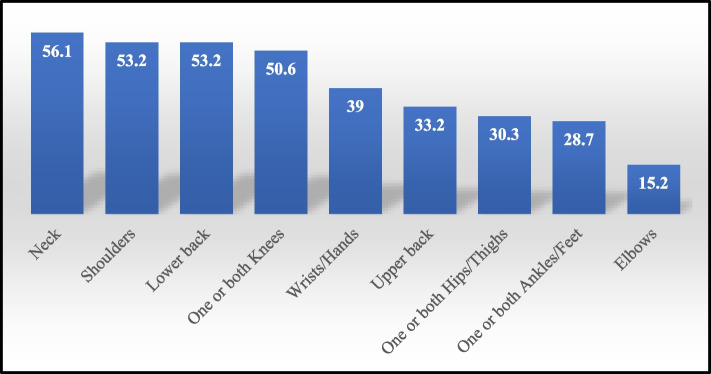
Prevalence of WRMSDs by body regions in the past 12- months among the studied school teachers: (N = 310)

The third category based on the self-reported prevalence of WRMSDs in the last 12 months included wrists/hands, upper back and hips/thighs affecting nearly or more than one-third of the teachers (39%, 33.2% and 30.3% respectively). The least affected body regions in terms of the 12-month prevalence were ankles/feet affecting 28.7% of the teachers, and elbow MSD that was the least reported disorder affecting only 15.2% of them (Fig. 1).

### Sociodemographic risk factors

Regarding the sociodemographic risk factors, there was no statistically significant difference (*p* = 0.71) between the mean age of teachers with WRMSDs (45.1 ± 5) in comparison to the mean age of teachers without WRMSDs (45.3 ± 5.4). There was a statistically significant difference (p ˂ 0.001) between teachers with and without WRMSDs as regards the gender. WRMSDs are more often prevalent in female teachers (81.5%) in comparison to male teachers (38.1%). Being a female teacher increased the risk of developing WRMSDs 2 times in comparison to the male teacher (PR = 2.14, CI = 1.67 – 2.75). No statistically significant difference was found between teachers with and without WRMSDs with respect to their marital status (*p* = 0.29).

A statistically significant difference (*p* = 0.009) was observed between the average BMI of teachers with WRMSDs (31.17 ± 6.37) compared to the average BMI of teachers without WRMSDs (29.28 ± 5.2). From all the teachers who suffered from WRMSDs, the highest prevalence of WRMSDs was among the obese teachers (55.6%). More than three quarters of the obese teachers (76.2%) suffered from WRMSDs. The obesity increased the risk of developing WRMSDs 1.33 times (PR = 1.33, CI = 1.04 – 1.7) (Table 1).

### Occupational risk factors

Regarding the occupational risk factors, no statistically significant difference (p = 0.45) was demonstrated between the average duration of teaching work experience of teachers with WRMSDs (21.5 ± 5.75) in comparison to that of teachers without WRMSDs (20.97 ± 6.14). A statistically significant difference by the number of students per classroom (p ˂ 0.001) was demonstrated between teachers with and without WRMSDs. Teachers with WRMSDs reported a higher average class size compared to those without WRMSDs (47.2 ± 10 vs 41.21 ± 11.13 respectively). Nearly three quarters of the teachers (72.7%) that had more than 35 students per class suffered from WRMSDs and had 1.63 times the risk of developing WRMSDs (PR = 1.63, CI = 1.23 – 2.16).

Teachers with WRMSDs differed significantly on the basis of their workload as described by the number of classes taught per week (*p* = 0.001) and the additional teaching of private groups after the end of the official scholastic day (*p* = 0.002) in comparison to teachers without WRMSDs. Three quarters of the teachers that had more than 20 classes in their weekly schedule (75.8%) or give study groups after the end of the school day (72.4%) developed WRMSDs. Teaching more than 20 classes per week and giving small study groups after the end of the official working hours increased the risk of reporting WRMSDs by 1.31 (PR = 1.31, CI = 1.11 – 1.54) and 1.32 (PR = 1.32, CI = 1.08 – 1.6) times respectively. Teachers with WRMSDs did not differ significantly in reference to the availability of appropriate and comfortable school furniture in comparison to their coworkers without WRMSDs (*p* = 0.66). However, it was observed that two thirds of the teachers who lack comfortable school furniture (67.8%) reported WRMSDs.

The frequency of the teachers who reported holding the neck in a forward bent posture (69.9% vs 38.7%, p ˂ 0.001), working with raised hands above the shoulder level (72.6% vs 58.1%, *p* = 0.008), standing (71.2% vs 57.8%, *p* = 0.02), sitting (76.8% vs 63.9%, *p* = 0.04), working in bent postures (72% vs 54.3%, *p* = 0.003) for prolonged periods was significantly higher in developing WRMSDs than the teachers that did not report adapting those postures for long periods. The risk of developing WRMSDs due to prolonged static head-down postures was 1.81 (CI = 1.15 – 2.83), repetitive working with raised hands above the shoulder level was 1.25 (CI = 1.05 – 1.49), long hours standing was 1.23 (CI = 1.02 – 1.48), sustained sitting was 1.2 (CI = 1.02 – 1.41) and recurrent leaning forward was 1.33 (CI = 1.08 – 1.63).

On the other hand, the number of the teachers who reported holding the neck in a backward bent posture (*p* = 0.51), holding the wrist bent (*p* = 0.052) and working in a twisted posture (*p* = 0.07) for prolonged durations did not reveal a significant difference in complaining from WRMSDs than the teachers that did not report adapting those postures for long durations (Table 2).

### Occupational psychosocial history of school teachers

Three quarters of the teachers (76.1%) reported that they had a work overload. 60.6% of them reported that they worked regularly under pressure of time. One third of the teachers stated that they hadn’t enough variety in work (33.2%) and did not enjoy mostly their work (35.5%). Half of the teachers (52.6%) reported the poor psychological support of the school administration. Only 15.2% of the teachers reported that they couldn’t count upon the psychological support of their colleagues when needed.

No statistically significant difference was noticed between teachers with and without WRMSDs as regards different work-related psychosocial factors such as having a high workload (*p* = 0.13), stressful working under pressure of time (*p* = 0.54), lack of enough variety in work (*p* = 0.28), poor psychological support from the colleagues (*p* = 0.84) and low job satisfaction (*p* = 0.7). However, more than two thirds of the teachers who reported high work demands (69.1%), perceived working regularly under pressure of time (68.1%), complained from monotonous work (70.9%), suffered from inadequate coworker support (68.1%) and stated that they were dissatisfied and did not enjoy their work (68.2%) developed WRMSDs.

The only psychological factor that has been found significantly associated (*p* = 0.007) with WRMSDs was the lack of enough supervisor’s psychological support at work. Nearly three quarters of the teachers (73.6%) that reported low supervisor support suffered from WRMSDs. They had 1.24 times the risk to develop WRMSDs (PR = 1.24, CI = 1.06 – 1.46) (Table 3).

**Table 3:** Comparison between teachers with and without WRMSDs as regards their work-related psychosocial history

		**All Teachers** **(*N* = 310)**		**Without WRMSDs** **(*N* = 103)**		**With** **WRMSDs** **(*N* = 207)**		**Statistical** **Test**	***P*- value**	**PR** **(95%** **CI)**
		**No**	**%**	**No**	**%**	**No**	**%**			
**Having too much to do** **at work**	Yes	236	76.1	73	30.9	163	69.1	χ^2^ = 2.34	0.13	
	No	74	23.9	30	40.5	44	59.5			
**Working regularly** **under pressure of time**	Yes	188	60.6	60	31.9	128	68.1	χ^2^ = 0.37	0.54	
	No	122	39.4	43	35.2	79	64.8			
**Having enough variety** **in work**	Yes	207	66.8	73	35.3	134	64.7	χ^2^ = 1.17	0.28	
	No	103	33.2	30	29.1	73	70.9			
**The supervision providing enough psychosocial support in work**	Yes	147	47.4	60	40.8	87	59.2	χ^2^ = 7.26	**0.007**	1.24 (1.06 – 1.46)
	No	163	52.6	43	26.4	120	73.6			
**Counting upon the psychosocial support** **of one of the colleagues** **if necessary**	Yes	263	84.8	88	33.5	175	66.5	χ^2^ = 0.04	0.84	
	No	47	15.2	15	31.9	32	68.1			
**Enjoying mostly the work**	Yes	200	64.5	68	34	132	66	χ^2^ = 0.15	0.7	
	No	110	35.5	35	31.8	75	68.2			

^Boldface values indicate statistical significance (^^*P* < 0.05)^

^***χ***^^***2*** Chi square test, *PR* Prevalence ratio, *CI* Confidence interval^

### Impact of WRMSDs on the QOL of school teachers

Concerning the mean values of the scores of the eight health-related QOL scales of the SF-36 as perceived by the studied teachers, the mean of the physical functioning scale was (83.7 ± 16.2), that of the role limitations due to physical heath scale was (44.7 ± 38.6), that of the role limitations due to emotional problems scale was (46.8 ± 41.7), that of the vitality scale was (45.5 ± 20.1), that of the mental health scale was (58.6 ± 21.2), that of the social functioning scale was (67.6 ± 29.4), that of the bodily pain scale was (64.9 ± 29.6) and that of the general health perception scale was (54.2 ± 19.4).

The negative impact of WRMSDs on the measures of QOL as perceived by the studied teachers was detected by comparing the mean SF-36 scores between the teachers with and without WRMSDs. The mean scores of the eight health-related QOL scales of the SF-36 were significantly lower (p ˂ 0.05) among the teachers with WRMSDs than those without WRMSDs. This finding reflected a lower physical and mental QOL of the teachers with WRMSD (Table 4).

**Table 4:** Comparison between teachers with and without WRMSDs as regards the impact of WRMSDs on the measures of QOL

Scale	All Teachers (*N* = 310)	Without MSDs (*N* = 103)	With MSDs (*N* = 207)	Statistical Test	*P*- value
	**Mean ± SD**	**Mean ± SD**	**Mean ± SD**		
**Physical functioning**	83.7 ± 16.2	92.48 ± 9.92	79.4 ± 17.01	t-test = 8.53	**˂0.001**
**Role limitations** **due to physical health**	44.7 ± 38.6	64.32 ± 38.29	34.9 ± 34.9	t-test = 6.77	**˂0.001**
**Role limitations** **due to emotional problems**	46.8 ± 41.7	59.55 ± 39.23	40.42 ± 41.59	t-test = 3.89	**˂0.001**
**Energy / Fatigue (Vitality)**	45.5 ± 20.1	54.66 ± 19.08	40.94 ± 18.99	t-test = 5.98	**˂0.001**
**Emotional well being** **(Mental health)**	58.6 ± 21.2	63.57 ± 20.86	56.08 ± 20.93	t-test = 2.97	**0.003**
**Social Functioning**	67.6 ± 29.4	79.61 ± 26.14	61.59 ± 29.22	t-test = 5.29	**˂0.001**
**Bodily Pain**	64.9 ± 29.6	94.85 ± 5.02	47.46 ± 19.72	t-test = 38.33	**˂0.001**
**General Health Perception**	54.2 ± 19.4	62.43 ± 19.31	50.12 ± 18.22	t-test = 5.49	**˂0.001**

^Boldface values indicate statistical significance (*P* < 0.05).^

^*SD* Standard deviation^

## Discussion

WRMSDs are named after the disorders that develop as a result of any problem related to the individual’s work [28]. They represent the most prevalent occupational disorders and their various risk factors have been thoroughly studied in different occupations [29–31]. Teaching profession is considered as one of the professions that is more vulnerable to develop WRMSDs [2, 6]. School teachers are more prone to suffer from MSP resulting either from their job demands such as prolonged sitting, standing and frequently supervising their students to monitor their learning progress and comprehensibility or from their working environment [5]. That’s why this study’s target population is teachers and only few studies focused on WRMSDs among Egyptian teachers.

The majority of the teachers participating in the present study were females aged between 40 and 50 years. Regarding the age range of the teachers, this finding is consistent with other studies where the average age of the teachers was above 40 years old [32, 33], while for the dominance of female teachers in our study population, this finding has been also observed in similar studies confirming that the teaching occupation is mainly predominated by females [1, 10, 23, 33]. Our results demonstrated that most of the teachers were overweight and obese which is similar to a Nigerian study [33]. This can be explained by the predominance of females in this study, and it is known that the prevalence of obesity is higher among the female gender [34]. In addition, the teaching profession is a sedentary occupation as it requires prolonged periods of static postures such as sitting and standing and teachers usually work for longer hours than required which restrict their physical activity [35].

### Prevalence of WRMSDs

Our results revealed that the *overall* self-reported prevalence of WRMSDs at any body region over the past 12 months was over 66% among the studied teachers. This finding is in line with a systematic review on MSDs among school teachers from different countries, that found that the prevalence of MSDs ranged between 39 and 95% [2]. This prevalence is also similar to that reported in previous studies among teachers in Estonia (66.7%) [36], Delhi, India (65.1%) [37], Saudi Arabia, Al-Jouf Region (68.5%) [24] and Saudi Arabia, Abha region (62.5%) [38]. It is lower than that was reported among teachers in Bolivia (86%) [17], Machakos, Kenya (85.1%) [39], Fiji (88.9%) [40], Nigeria (70.2%) [33], Hail, Saudi Arabia (87.3%) [25] and Chile (88.9%) [41]. However, it is higher in comparison with that reported among teachers in Brazil (55%) [1], Turkey ((51.4%) [8], (36%) [42]) and Terengganu, Malaysia (40.1%) [43].

The relatively high overall self-reported prevalence of WRMSDs among Egyptian school teachers might be partly explained by their stressful working conditions especially in public schools where they face greater work demands and are provided with poor facilities. Classrooms, in a developing country as Egypt, are overcrowded. The classroom density may be as high as 45 to 55 students per class. To cope with high classroom densities, the ministry of education attempted to divide the scholastic day into two shifts, morning and evening shifts, in order to accommodate all the children which results in overburdening of teachers [44]. Teachers also suffer from low income forcing them to teach private study groups after the scholastic hours.

*Neck* pain was the most prevalent MSP in the last 12 months in the current study. This high prevalence may be explained by the frequent adoption of prolonged ‘head down’ positions as when reading, preparing lessons, and marking of assignments and examination papers and may be also attributed to the significant use of neck extension postures for long durations while writing on or reading from the board [11, 14].

A similar prevalence of neck pain has been demonstrated in previous studies such as 56.8% in Saudi Arabia [5], 53.52% in India [45], 53.3% in Machakos, Kenya [39], 56.2% in Chile [41] and 57.9% in Nigeria [33]. However, this figure is better than that has been found in previous studies such as 66.7% in Hong Kong [14], 61.3% in Iran [46] and 75.5% in Kuala Lumpur [47]. On the other hand, a lower prevalence has been reported in previous studies such as 47.2% in Bolivia [17], 22.6% in Terengganu, Malaysia [43], 48.5% in Fiji [40] and 36.25% in Hail, Saudi Arabia [25].

*Shoulder* pain was the second most common musculoskeletal complaint among teachers in this study. This finding is in line with the literature as shoulder MSDs have been found to be one of the three most commonly MSDs affecting the school teachers [7, 11], with a higher prevalence rate in comparison with the other occupational groups [1]. High prevalence of complaints at the shoulder joint may be attributed to working with hands above the shoulder level for prolonged durations as while writing on the board and to repetitive movements with arms, hands or fingers several times per day [45].

This result is similar to previous research conducted in Turkey (55.9%) [9], Botswana (52.5%) [6], China (52.29%) [15] and Hail, Saudi Arabia (53.39%) [25]. It is better than the rate reported among teachers in Saudi Arabia (60.6%) [5], Kuala Lumpur (80.1%) [47] and Enugu, Nigeria (62.3%) [33]. However, it is higher than the rate of other studies that were conducted in Bolivia (34.6%) [17], Terengganu, Malaysia (22.2%) [43], Fiji (46.6%) [40] and Saudi Arabia, Abha region (47.9%) [38].

*Low back* pain was also found to be the second most common musculoskeletal disorder among teachers in this study. The high prevalence of self-reported LBP may be due to improper techniques of carrying heavy teaching materials to the class like workbooks, examination sheets, projectors and lab instruments, prolonged standing while teaching and supervising the students, poor postures while sitting during preparing lessons, marking homework, grading examinations and working on computer, frequent bending or recurrent twisting as turning from the board to the classroom and back again, climbing up and down the stairs and lack of appropriate chairs with comfortable back support [8, 10, 39, 45, 48–50]. LBP is also a psychosocial health problem affected by the psychosocial factors at the school environment since teachers usually work in a stressful environment due to the deficiency of educational resources, limited rewards, unpredictable students` or their parents’ behaviour and inadequate support from the school administrative staff and colleagues [51].

This prevalence is consistent with the findings of similar studies conducted in Botswana (55.7%) [52], in Machakos, Kenya (58.6%) [39], Saudi Arabia, Abha region (59.2%) [38] and Chile (57.5%) [41]. It is lower than what has been reported in similar studies in Turkey (74.9%) [9], Iran (71.9%) [46], Saudi Arabia, Al-Jouf Region (68.4%) [24] and Hail, Saudi Arabia (62.55%) [25]. However, it is higher than what has been reported in similar studies in China (47.14%) [15], India (33.8%) [45], Terengganu, Malaysia (25%) [43], Fiji (45.4%) [40] and Enugu, Nigeria (49%) [33].

*Knee* pain was the third musculoskeletal complaint among teachers in this study. Standing for long hours during teaching and other school activities increases load on the knees joints which can interpret this high prevalence of knee pain among teachers [37, 45]. The prevalence of knees MSDs is consistent with that reported by school teachers in India (55.2%) [53], Machakos, Kenya (57.6%) [39] and Saudi Arabia, Al-Jouf Region (58.6%) [24]. It is higher than the one reported among teachers in Bolivia (37.5%) [17], Saudi Arabia, Abha region (43.3%) [38], Fiji (21.8%) [40], Terengganu, Malaysia (28.8%) [43], Enugu, Nigeria (39.3%) [33] and Hail, Saudi Arabia (41.04%) [25]. However, it is lower than that reported by teachers in Saudi Arabia (63.2%) [5] and Kuala Lumpur (88%) [47].

Similar prevalence of *wrists/hands* MSDs was reported by teachers in Saudi Arabia (40.5%) [5], Machakos, Kenya (42.4%) [39], Enugu, Nigeria (48.6%) [33], Hail, Saudi Arabia (30.3%) [25] and Chile (45.1%) [41]. The prevalence of wrists/hands pain in this study is higher when compared with that found in other studies conducted in Bolivia (25.7%) [17], Saudi Arabia, Al-Jouf Region (14.4%) [24], Terengganu, Malaysia (9.9%) [43] and Fiji (12.2%) [40]. On the other hand, it is less in comparison with the one that was found in India teachers (66.6%) [53].

*Upper back* pain prevalence in this study is consistent with the findings of previous research carried out in Turkey (36.9%) [8], India (39.43%) [45], Bolivia (35.8%) [17] and Hail, Saudi Arabia (33.5%) [25]. A higher upper back pain prevalence was found in Botswana (52.6%) [6], Machakos, Kenya (42.4%) [39], Enugu, Nigeria (47.4%) [33] and Chile (45.1%) [41]. However, a lower upper back pain prevalence was found in Turkey (8%) [42], Delhi, India (10.9%) [37], Fiji (25.6%) [40], Terengganu, Malaysia (26.4%) [43].

*Hips/Thighs* pain prevalence in the present study is similar to the estimated prevalence from similar studies in Bolivia (31.9%) [17], Machakos, Kenya (25.8%) [39], Chile (28.8%) [41] and Hail, Saudi Arabia (37.05%) [25]. It is higher than the one reported by teachers in Botswana (18.2%) [6], China (16.57%) [15], Delhi, India (15.4%) [37], Terengganu, Malaysia (18.4%) [43] and Fiji (9.5%) [40]. However, it is lower than that reported by teachers in Enugu, Nigeria (45.3%) [33].

*Ankles/Feet* prevalence is nearly equal to the one reported among teachers in Turkey (29.5%) [9], Delhi, India (26.3%) [37], Terengganu, Malaysia (32.5%) [43], Enugu, Nigeria (30.8%) [33] and Hail, Saudi Arabia (31.5%) [25]. It is lower when compared to what has been demonstrated in Iran (46.8%) [46], Saudi Arabia (56%) [5] and Machakos, Kenya (53%) [39]. However, it is higher when compared to what has been found in Al-Khobar, Saudi Arabia (12.3%) [11], Turkey (13%) [42] and Fiji (15.3%) [40].

*Elbow* pain prevalence of the current study is similar to the results of other studies conducted in Botswana (13.3%) [6], Bolivia (12.3%) [17], Delhi, India (18.5%) [37], Terengganu, Malaysia (10.4%) [43] and Hail, Saudi Arabia (16.3%) [25]. This result is lower than other studies in Saudi Arabia (42%) [5] and Machakos, Kenya (25.2%) [39]. However, it is higher than other studies in Turkey ((8%) [8], (5%) [42]) and Fiji (4.6%) [40].

The observed difference in the prevalence rates of WRMSDs at any body segment among school teachers from different countries may be related to different aspects of their lifestyle and their working conditions such as the teaching hours in day, the student–teacher ratio, the usage of computers in the teaching process and the other facilities provided for them at schools [1, 39]. It may be also explained by the cultural, social, and economic differences between Egypt and the other mentioned countries, the provision of health services and the availability of educational and training programs on different workplace hazards and ergonomic issues [16, 54]. Methodological differences between the studies as differences in the study design, the study population, the sample size and the defined prevalence periods [42] may be another factor contributing to the observed differences in the prevalence rates.

### Sociodemographic risk factors

*Age* of the teachers had no significant relationship with WRMSDs in our study. In the literature, conflicting results have been demonstrated in the association between age and WRMSDs. Our result agrees with previous studies that did not find a significant association between age and the development of WRMSDs among school teachers [9, 12, 55, 56]. On the other hand, a positive association was established between age and the development of WRMSDs in the different body regions among teachers in other studies [8, 10, 11, 14, 24, 33, 39, 49, 54]. While age has been found positively associated with WRMSDs, there were inconsistent research findings between the studies. Some studies reported that the prevalence of WRMSDs increased with advancing age, whereas others stated that younger teachers were more likely to experience MSP [57].

The lack of a significant association between increase in age and development of WRMSDs among teachers in our study is concerning because it seems that younger teachers also tend to experience WRMSDs early in their career as older teachers since a high prevalence of WRMSDs was reported in all age groups. This can be explained by the fact that younger teachers may be asked for more duties and activities at the beginning of their career [1]. Facing high work demands, they may not adapt well to their new working environment and consequently increase their physical and psychological stress leading to early experience of MSDs [14]. It can be also explained by the healthy worker effect that can lead to selection bias resulting from the presence of healthy teachers in all age groups and the absence of teachers who took a sick leave or were early retired because of a disabling MSP and consequently masks the true effect of age on the development of WRMSDs [48]. Moreover, the usage of the self-reporting NMQ for assessment of MSP might have a possibility of recall bias due to the long recall period (past 12 months) and subjectivity in reporting of MSP which is not based on an objective clinical diagnosis by a specialist but may be influenced by the negative perspective of the teachers towards their health and work conditions. These factors might lead to under or overreporting of WRMSDs [6, 10, 50].

The current study demonstrated that *female gender* was significantly associated with the development of WRMSDs. This finding is supported by the results of various studies that detected a significant higher prevalence of MSDs among female teachers in comparison to their male counterparts that suggested a positive relationship between female gender and the occurrence of WRMSDs in the teaching profession [1, 2, 6, 8–10, 12, 14, 25, 49, 58].

This gender difference may be attributed to several factors. First, females have a higher risk of developing MSP since the teaching profession is primarily dominated by females [1, 23, 57]. Second, female teachers were found to suffer more emotional exhaustion and work-related stress than their male colleagues [5]. Third, females are known to report pain more frequently than males as females may have more pressure from their family and career responsibilities or they may have lower pain thresholds and different traditions for the time and the way in which they report pain [7, 8, 14]. Fourth, women are more involved in heavy housework daily in comparison to men who have less participation in household tasks [10]. Fifth, it was found that men had more regular physical exercise practice compared to women [6, 9, 49]. Sixth, women were found to have higher BMI in previous studies [6, 49]. Finally, menstruation, pregnancy and osteoporosis may be other possible reasons [2].

The present study did not establish a relationship between *marital status* of the teachers and the occurrence of WRMSDs. This finding agrees with a study conducted among Nigerian school teachers [33].

Our results revealed that *BMI* had a significant relationship with WRMSDs. This finding is consistent with similar studies in different countries that showed a significant association between the BMI of the studied teachers and the development of WRMSDs [10–12, 38, 40, 43]. The reason behind this association that overweight and obesity increase the mechanical stress on the joints resulting in MSP [7]. The individual with high BMI needs more effort to do the same work as an individual with normal BMI leading to more muscle activity and muscle injury resulting in MSDs [28].

### Occupational and psychosocial risk factors

The present study did not demonstrate a significant association between the *length of employment* of the studied teachers and WRMSDs as it was found that teachers who reported WRMSDs and those who did not report WRMSDs had nearly the same mean of the years spent in the teaching profession. Contradicting findings exist in the literature concerning the association of the length of employment and WRMSDs in the teaching profession [57]. Our result is consistent with similar studies that did not find a significant relation between the teaching experience and WRMSDs [5, 9, 58]. However, our result disagrees with the results of previous research that reported a positive association between years of teaching experience and developing WRMSDs [1, 10, 11, 14, 24, 42, 54].

While the length of employment has been reported to be positively associated with WRMSDs, the research results were conflicting in the literature. Some studies found a higher prevalence of WRMSDs among school teachers with a longer length of employment due to the effect of the aging process, the cumulative effect of the workloads over years on the musculoskeletal system of the teachers [58] and the longer time of exposure to work-related risk factors with a higher chance of developing WRMSDs over time [14, 24]. Conversely, other studies detected that the newly employed teachers were more likely to develop musculoskeletal pain disorders [2] as they are still adapting to the new working environment and their musculoskeletal system is easily influenced by the physical and psychological stresses [59]. It has been also suggested that newly married female teachers and having small children were more likely to report MSP [8].

The *number of students* in the classroom had a positive association with WRMSDs in the present study. This finding is consistent with previous research that found a significant association between musculoskeletal problems among teachers and increasing number of students in the classroom [1, 33]. The reason of this association may be that the increasing number of students requires additional duties and activities from the teacher such as correction of students’ assignments and activities and examination papers and more attention and supervision [1].

The *number of classes* taught by the teachers per week showed a significant association with the development of WRMSDs in the current study. Our result agrees with the results of other studies that reported a relationship between the increase in the number of classes per week [12, 24] or the increase in the working hours [1, 55] or a high workload [14, 58] and musculoskeletal pain disorders among teachers. The possible explanation for this association that increase in the workloads by long working hours will increase the stress on the musculoskeletal system of the teachers resulting from awkward postures, prolonged standing and sitting with limited time for recovery. This cumulative effect of work stress on the muscles and joints will accelerate the occurrence of WRMSDs [58].

This study did not establish an association between *uncomfortable school furniture* with the prevalence of WRMSDs. This result disagrees with similar studies that established a positive association between inappropriate and uncomfortable school furniture and musculoskeletal pain disorders [1, 10, 24]. If the school furniture is inappropriate in size and shape for the teachers or without adequate back and hands support, they will be obliged to adapt to awkward positions unfavorable for their musculoskeletal system. For example, low chairs will result in excessive flexion of the trunk and the hip and knee joints while reading or writing texts [14].

In the present study, many adapted work-related *postures* by the teachers were found to be significantly related to the occurrence of WRMSDs such as holding the neck in a forward bent posture for prolonged periods, working with hands above shoulder level for long periods, prolonged standing or sitting and working in bent postures for long durations. The above findings are consistent with many studies in the literature that demonstrated a significant relationship between various physical factors and the increased occurrence of MSDs among teachers. In a Chinese study, prolonged standing, sitting and static postures were strongly related with neck, shoulder and low back pain among teachers [10]. In an Indian study, the self- reported physical risk factors by teachers were holding hands above shoulder level and working in bent postures for long periods [45]. In a study conducted in Fiji, it was found that the significant MSP increasing factors among secondary school teachers were prolonged sitting and standing and bending positions [40].

Teaching is considered as a physically demanding occupation that requires the adoption of repetitive static, poor and awkward working positions for prolonged durations while performing the different occupational activities. It appears that teachers spend much of their time in different unfavorable working conditions and are exposed to a high occupational postural and physical loading in their daily classroom routines that has been considered the most important predisposing risk factor for developing musculoskeletal pain disorders [1, 14, 58]. This physical overload produces a biomechanical stress on the musculoskeletal structures and consequently increases the risk of musculoskeletal injuries among school teachers [55, 60].

The significant use of static *head-down posture* for several hours has been demonstrated as a major risk factor for neck, shoulder and upper limb pain among teachers in various studies [8, 14, 16, 54, 56]. The reason might be that sustained flexion of the head increases the load and tension on the posterior cervical structures causing muscle stiffness and discomforts [8]. The repetitive overhead *writing on board* with hands elevated above shoulder level has been found significantly associated with neck and shoulder injuries in many studies [6, 8, 10, 14, 16, 53]. Working with raised arms for a long time to write on the board causes strain and tension on the cervicobrachial structures resulting in neck, shoulder and upper limb pain [8].

*Prolonged standing* during work had a significant relationship with MSP among teachers in previous studies [38, 56]. This might be attributed to the limited spinal movement with extended hours of standing, which increases the load and the strain on the lumbar spine and tissues and consequently causes MSP [2]. *Prolonged sitting* was positively associated with a higher prevalence of MSDs among teachers in other studies [10, 39]. The risk of MSP increases when teachers are provided with uncomfortable furniture and have to sit awkwardly without adequate support which strains their lower back [39].

The current study did not demonstrate a significant association between the different psychosocial risk factors such work overload, working under pressure of time, monotonous work, low job satisfaction, inadequate colleagues’ psychological support and the development of WRMSDs among the studied teachers. This result is supported by similar studies that did not find a positive association between psychological factors and MSP among teachers like high workloads and job demands [48], job dissatisfaction [6, 50], low coworker support [6, 50], and low supervisor support [6, 49]. Conversely, this result disagrees with other studies in the literature that have indicated that psychological factors like high work demands, low job control, perception of a high stress level at work, job dissatisfaction, monotonous work, lack of social support had a significant association with musculoskeletal symptoms among school teachers [2, 13, 14, 57, 58]. The observed discrepancy between the studies regarding the association between the psychological factors and WRMSDs can be explained by the use of different methods for the evaluation of psychological factors that were usually subjective and self- reported as in this study [50].

In this research, the only psychological factor that has been found significantly associated with WRMSDs was the lack of enough supervisor’s psychological support at work. This result agrees with previous studies. In Japan and Malaysia, teachers complaining from low supervisor support were more prone to develop neck and shoulder pain [48, 58]. In Kenya, poor supervisor support was the only psychological factor associated with a higher odds of LBP [50].

### Impact of WRMSDs on the QOL

Our results revealed that the scores of the 8 health-related QOL scales of SF-36 were significantly lower among the teachers having WRMSDs, which reflects that the teachers reporting MSP have a lower physical and mental QOL than the ones without MSP. WRMSDs had a negative impact on the QOL of the studied teachers affecting their wellbeing and probably the teaching process itself and making the teachers seeking for medical treatment to relief the pain. Similar findings have been reported in the literature [6, 11, 14, 25, 46, 60].

This result agrees with a Turkish study that found that teachers having MSP had significantly lower scores on five of QOL scales of SF-36 [9]. In another Turkish study, the scores of the physical component scale, the bodily pain and the vitality domains were significantly lower among the teachers reporting musculoskeletal symptoms, indicating the negative impact of WRMSDs especially on the physical components of QOL [42]. In a Chilean study, significant differences were observed between teachers reporting MSDs in over six body regions and those reporting MSDs in six or fewer body regions on six of QOL scales, and a significant association was observed between the highest prevalence MSDS cases and the lowest scores in the physical and mental components measurements for QOL. Therefore, teachers having higher rates of MSDs have their physical and mental QOL affected [41].

## Study limitations

*The present study had a number of limitations which included:* Firstly, the cross-sectional design of our study allowed only to identify the association between the risk factors under study and the occurrence of WRMSDs among school teachers. However, it was difficult to establish any causality and effect inferences since the temporal relationship between the independent variables of interest and the outcome couldn’t be determined. Despite this limitation, this type of study is still valuable in the identification of the various factors most vulnerable to WRMSDs. Secondly, it was also susceptible to the healthy worker bias. This means that only healthy and working teachers who volunteered to participate in this study were assessed, while those who took a sick leave or were early retired because of a disabling MSP were excluded. This type of bias might result in underestimation of the prevalence rate of WRMSDs among teachers. Thirdly, the data were obtained only from teachers working in governmental schools. Lastly, all the variables were assessed by self-reporting measures. Although the used instruments such as the Nordic questionnaire and SF-36 survey were validated internationally and widely adopted, there might be a possibility of recall bias due to the nature of those retrospective surveys and the long recall period, for example the teachers might not correctly remember the presence of WRMSDs in the past 12 months. Moreover, the collected data were subjective, not based on an objective clinical diagnosis by a specialist or an ergonomic analysis for the workplace and might be influenced by the negative perspective of the teachers towards their health and work conditions. These factors might lead to under or overestimation of the outcome.

## Conclusion and recommendations

In conclusion, WRMSDs were a highly prevalent occupational health problem among school teachers in Cairo, Egypt since two thirds of them had experienced MSP at any body region over the past 12 months. The body regions with the highest prevalence rates of musculoskeletal trouble were the neck, shoulders, lower back and knees. The least affected body part was the elbow MSD. They had a negative impact on the physical and mental components of QOL of the teachers with WRMSDs as reflected by their significantly low mean scores on the different QOL scales of the SF-36 survey compared to those without WRMSDs.

Different individual, occupational and psychosocial factors had been shown to be significant predictors for the occurrence of WRMSDs among the studied teachers reflecting their complex nature and multifactorial etiology. Female gender and BMI were among the individual factors positively associated with WRMSDs. Occupational factors such as the number of students per classroom, the number of classes per week, teaching study groups after the end of the scholastic day, awkward postures as head-down posture for prolonged periods, working with hands above shoulder level for long periods, working in bent postures for long durations, prolonged standing and sitting increased the odds of WRMSDs among teachers. The lack of enough supervisor’s psychological support at work was the only significant psychological risk factor for WRMSDs.

The findings highlighted the need to develop educational programs, to plan, design and implement effective preventive and therapeutic strategies and to follow the ergonomic rules to reduce the stress imposed on the teachers’ musculoskeletal system, the prevalence and the progression of WRMSDs among them and their drawbacks. They also would help to promote their health and occupational well-being and to ensure a better QOL for this professional group.

## Data Availability

The datasets generated and/or analysed during the current study are not publicly available to ensure the confidentiality of the data as mentioned in the consent form and according to the ethics approval and not to disclose identifying information that could compromise the privacy of the participants. However, they are available in an anonymized form from the corresponding author on reasonable request.

## Abbreviations

WRMSDs: Work-related musculoskeletal disorders
MSDs: Musculoskeletal disorders
LBP: Low back pain
BMI: Body mass index
MSP: Musculoskeletal pain
QOL: Quality of life
DMQ: Dutch Musculoskeletal Questionnaire
NMQ: Nordic musculoskeletal questionnaire
SF-36: Short Form-36 Health Survey
HTI: Health transition item
